# Dried blood spot characterization of sex‐based metabolic responses to acute running exercise

**DOI:** 10.1002/ansa.202200039

**Published:** 2023-02-05

**Authors:** Francesca Cendali, Angelo D'Alessandro, Travis Nemkov

**Affiliations:** ^1^ Department of Biochemistry and Molecular Genetics University of Colorado Anschutz Medical Campus Aurora Colorado USA

**Keywords:** bioenergetics, dried blood spot, exercise, fatty acid oxidation, glycolysis, lipidomics, metabolomics, running

## Abstract

Metabolomics and lipidomics techniques are capable of comprehensively measuring hundreds to thousands of small molecules in single analytical runs and have been used to characterize responses to exercise traditionally using venipuncture‐produced liquid samples. Advanced microsampling devices offer an alternative by circumventing the requirement to maintain frozen samples. This approach combines a microneedle puncture for blood draw with microfluidic sample collection onto a dried carrier and has thus far been employed for targeted measurements of a few analytes. To demonstrate the utility of advanced dried microsampling to characterize metabolomic and lipidomic changes during exercise, we obtained samples before and after a 2‐mile run from twelve (8 male, 4 female) healthy volunteers with various ranges in activity levels. Results highlighted significant changes in whole blood levels of several metabolites associated with energy (glycolysis and Tricarboxylic Acid cycle) and redox (Pentose Phosphate Pathway) metabolism. Lipid changes during this same period were individualized and less uniform. Sex‐based differences in response to running highlighted reliance on carbohydrate or fat substrate utilization in males or females, respectively. The results presented herein illustrate the ability of this approach to monitor circulating metabolome and lipidome profiles from field sampled blood in response to exercise.

## 1 INTRODUCTION

Non‐communicable diseases including cardiovascular disease, as well as the metabolic factors that etiologically contribute to their onset (e.g., obesity and diabetes), currently represent the global leading cause of death.^1^ Sedentary behavior is a conduit for the development of metabolic derangements that contribute to the development of cardiovascular diseases^2^ and obesity,^3^ the latter of which has nearly tripled since 1975 and accounts for one third of the 1.9 billion adults worldwide who are reported to be overweight.^4^ As such, understanding how exercise rewires metabolism to prevent and mitigate obesity and associated cardiovascular disease is becoming an increasingly critical research endeavor.

Mass spectrometry‐based metabolomics and lipidomics approaches have revived the field of sports metabolism,^5^ by expanding classic concepts including the anaerobic or lactate threshold (equilibrium between synthesis and clearance^6^) and substrate utilization,^7^ with the ability to monitor thousands of small molecule analytes in a single comprehensive analysis. Metabolomics studies in the context of endurance, resistance, or high‐intensity bouts of exercise have been described in the literature.^8^, ^9^, ^10^, ^11^, ^12^ Most of these studies have been performed under well‐controlled laboratory settings, focusing on exercise and environmental characteristics (e.g., high‐intensity concentric‐eccentric exercise^13^ under hypoxia^14^ or circadian regulation of exercise in animal models).^15^ However, limited literature is currently available on the impact of exercise in the field under real world conditions (i.e., outside a controlled laboratory setting).

A common limitation encountered during the design of these metabolomics studies pertains to the sample collection. Options are often limited to venipuncture, which requires trained phlebotomists and represents a logistical hurdle to sample collection in the field. Recently, we and others reported on the use of microcapillary‐based touch activated (TAP) devices for blood sampling^16^ and its utility in characterizing whole blood metabolomes of elite professional cyclists.^10^ TAP sampling, however, is limited by the requirement for freezing liquid whole blood samples to ‐80°C immediately after collection and throughout storage prior to mass spectrometry‐based characterization. As such, methods that provide dried immobilized blood samples offer an opportune mechanism to implement metabolomics and lipidomics for the characterization of outdoor sports training and performance monitoring (reviewed in^17^). In addition to dried blood spot collection onto filter cards,^18^ volumetric absorptive microsampling (VAMS, Neoteryx, Torrance, CA) that also utilizes a lancet for blood draw has been implemented for targeted mass spectrometry‐based quantification of small molecule metabolites^19^ and lipids.^20^ Additional advanced dried sample devices that couple microcapillary‐based sampling to dried blood spot collection such as the TASSO‐M20 (Tasso, Inc., Seattle, WA) offers an alternative for ambient‐stable sample collection. As this device is capable of quantifying growth hormone^21^ and other illicit doping substances,^22^, ^23^, ^24^, ^25^ it has been welcomed by the World Antidoping Agency^26^ and is on track for integration into the current anti‐doping testing system.^27^ However, a metabolomics characterization is limited^28^ to pilot explorations for clinical purposes and—to the best of our knowledge—never applied to assess the impact of exercise in field testing, which is the focus of the present study.

Here we used an advanced microcapillary‐based blood sampling device to collect dried blood samples from 12 volunteers at baseline and after a 2‐mile run in the field. We then applied a high‐throughput multi‐omics approach to analyze these samples, showing proof of concept for metabolomics and lipidomics analysis of field samples. Additional samples were collected 24 hours after completion of the trial to assess the feasibility of multi‐omics‐based characterization of inter‐subject heterogeneity in the recovery phase from the same exercise regimen.

## 2 MATERIALS AND METHODS

*Acute running test*: Twelve recreational athletes (*n* = 8 males and *n* = 4 females) voluntarily participated in this trial (demographic details provided in Table 1). After a 5‐minute warm‐up, subjects ran the same 2‐mile course guided at self‐pace, though all subjects started together. All study procedures were conducted in accordance with the Declaration of Helsinki.

**TABLE 1:** Subject information including means and standard deviations for each value

Subject ID	Gender	Age (Years)	Height (m)	Weight (kg)	BMI (kg/m2)	Time (min:sec)
S1	M	43	1.78	72.6	23.0	14:37
S2	M	30	1.88	70.3	19.9	14:03
S3	F	36	1.63	49.0	18.5	14:51
S4	M	38	1.78	68.0	21.5	14:47
S5	M	29	1.80	77.1	23.7	14:27
S6	M	27	1.78	68.0	21.5	11:54
S7	M	36	1.85	74.8	21.8	11:55
S8	M	25	1.63	64.9	24.5	17:38
S9	M	35	1.75	74.8	24.4	14:40
S10	F	28	1.60	57.6	22.5	14:43
S11	F	25	1.70	54.4	18.8	12:03
S12	F	30	1.63	48.5	18.4	17:16
Mean		32	1.73	65.00	21.5	14:24
SD		5	0.09	9.74	2.1	1:45

*Dried blood sampling*: Dried whole blood samples were obtained immediately before and after a 2‐mile run (*n* = 12), and 24 hours after the post‐run sample from a subset of male subjects (*n* = 7) using the TASSO‐M20 device (Tasso, Inc, Seattle, WA, USA) according to manufacturer's instructions. The device collects whole blood into 4 individual dried blood samples (DBS) containing 17.5 µL each after which additional blood collects at the bottom of the sample pod thereby enabling visual confirmation that each of the spots are saturated. Furthermore, volumetric absorption ensures equivalent volumes of whole blood are uniformly collected in each spot, thus mitigating the “hematocrit effect”.^29^ After collection, samples were dried for 1 hour under ambient conditions, and upon confirmation that all spots were dry, were sealed in the provided airtight containment with a desiccant pouch. All samples were stored together at room temperature for 24‐48 hours depending on time of collection (pre‐test, immediately after or 24 h post‐exercise) before extraction for metabolomics or lipidomics analysis.

*Sample Preparation for LC‐MS*: Prior to metabolomics or lipidomics analysis, individual DBS from each donor were placed in 1.5 ml tubes for either metabolite or lipid extraction. DBS were suspended in 180 µL of water/methanol (50:50 v/v) for metabolite extraction or 180 µL of isopropanol/methanol (50:50 v/v) for lipid extraction. Suspensions were sonicated for 20 minutes at room temperature, subsequently incubated at 4°C for 30 minutes, and then centrifuged for 10 minutes, 18213 g, 4°C. Supernatants were isolated for LC‐MS.

*UHPLC‐MS data acquisition and processing*: Analyses were performed as previously published.^30^, ^31^, ^32^ Briefly, the analytical platform employs a Vanquish UHPLC system (Thermo Fisher Scientific, San Jose, CA, USA) coupled online to a Q Exactive mass spectrometer (Thermo Fisher Scientific, San Jose, CA, USA). Polar metabolite extracts were resolved in singlicate over a Kinetex C18 column, 2.1 × 150 mm, 1.7 µm particle size (Phenomenex, Torrance, CA, USA) equipped with a guard column (SecurityGuard™ Ultracartridge—UHPLC C18 for 2.1 mm ID Columns, Phenomenex, Torrance, CA, USA) using an aqueous phase (A) of water and 0.1% formic acid and a mobile phase (B) of acetonitrile and 0.1% formic acid for positive ion polarity mode, and an aqueous phase (A) of water:acetonitrile (95:5) with 1 mM ammonium acetate and a mobile phase (B) of acetonitrile:water (95:5) with 1 mM ammonium acetate for negative ion polarity mode. The column was equilibrated at 5% B, and upon injection of 10 µl of extract, samples were eluted from the column using the solvent gradient: 0.5‐1.1 min 5‐95% B at 0.45 mL/min; hold at 95% B for 1.65 min at 0.45 mL/min, and then decrease to 5% over 0.25 min at 0.45 ml/min, followed by a re‐equilibration hold at 5% B for 2 minutes at 0.45 ml/min. The Q Exactive mass spectrometer (Thermo Fisher Scientific, San Jose, CA, USA) was operated independently in positive or negative ion mode, scanning in Full MS mode (2 µscans) from 60 to 900 m/z at 70000 resolution, with 4 kV spray voltage, 45 sheath gas, 15 auxiliary gas, AGC target = 3e6, maximum IT = 200 ms. Non‐polar lipid extracts were resolved over an ACQUITY HSS T3 column (2.1 × 150 mm, 1.8 µm particle size (Waters, MA, USA) using an aqueous phase (A) of 25% acetonitrile and 5 mM ammonium acetate and a mobile phase (B) of 90% isopropanol, 10% acetonitrile and 5 mM ammonium acetate. The column was equilibrated at 30% B, and upon injection of 10 µl of extract, samples were eluted from the column using the solvent gradient: 0‐9 min 30‐100% B at 0.325 mL/min; hold at 100% B for 3 min at 0.3 mL/min, and then decrease to 30% over 0.5 min at 0.4 ml/min, followed by a re‐equilibration hold at 30% B for 2.5 minutes at 0.4 ml/min. The Q Exactive mass spectrometer (Thermo Fisher) was operated in positive ion mode, scanning in Full MS mode (2 µscans) from 150 to 1500 m/z at 70000 resolution, with 4 kV spray voltage, 45 sheath gas, 15 auxiliary gas. When required, dd‐MS2 was performed at 17500 resolution, AGC target = 1e5, maximum IT = 50 ms, and stepped NCE of 25, 35 for positive mode, and 20, 24, and 28 for negative mode. Calibration was performed prior to analysis using the Pierce™ Positive and Negative Ion Calibration Solutions (Thermo Fisher Scientific).

*Data analysis*: Acquired data was converted from raw to mzXML file format using Mass Matrix (Cleveland, OH, USA). Samples were analyzed in randomized order with a technical mixture injected after every 10 samples to qualify instrument performance. Metabolite assignments were performed using accurate intact mass (sub‐10 ppm), isotopologue distributions, and retention time/spectral comparison to an in‐house standard compound library (MSMLS, IROA Technologies, NJ, USA) using MAVEN (Princeton, NJ, USA).^33^ Lipidomics data were analyzed using LipidSearch 4.0 (Thermo Scientific), which provides lipid identification on the basis of accurate intact mass, isotopic pattern, and fragmentation pattern to determine lipid class and acyl chain composition. Graphs, heat maps and statistical analyses (either T‐Test or ANOVA), multivariate analyses including Principal Component Analysis (PCA), Partial Least Squares‐Discriminant Analysis (PLS‐DA), hierarchical clustering analysis (HCA), and metabolite pathway enrichment analysis were performed using MetaboAnalyst 5.0.^34^ Individual metabolite graphs were plotted with GraphPad Prism 9 (GraphPad Software Inc., La Jolla, CA, USA). Pathway graphs were prepared on BioRender.com.

## 3 RESULTS AND DISCUSSION

### 3.1 Dried blood spot metabolomics reveals metabolic changes in response to acute running

**FIGURE 1:**
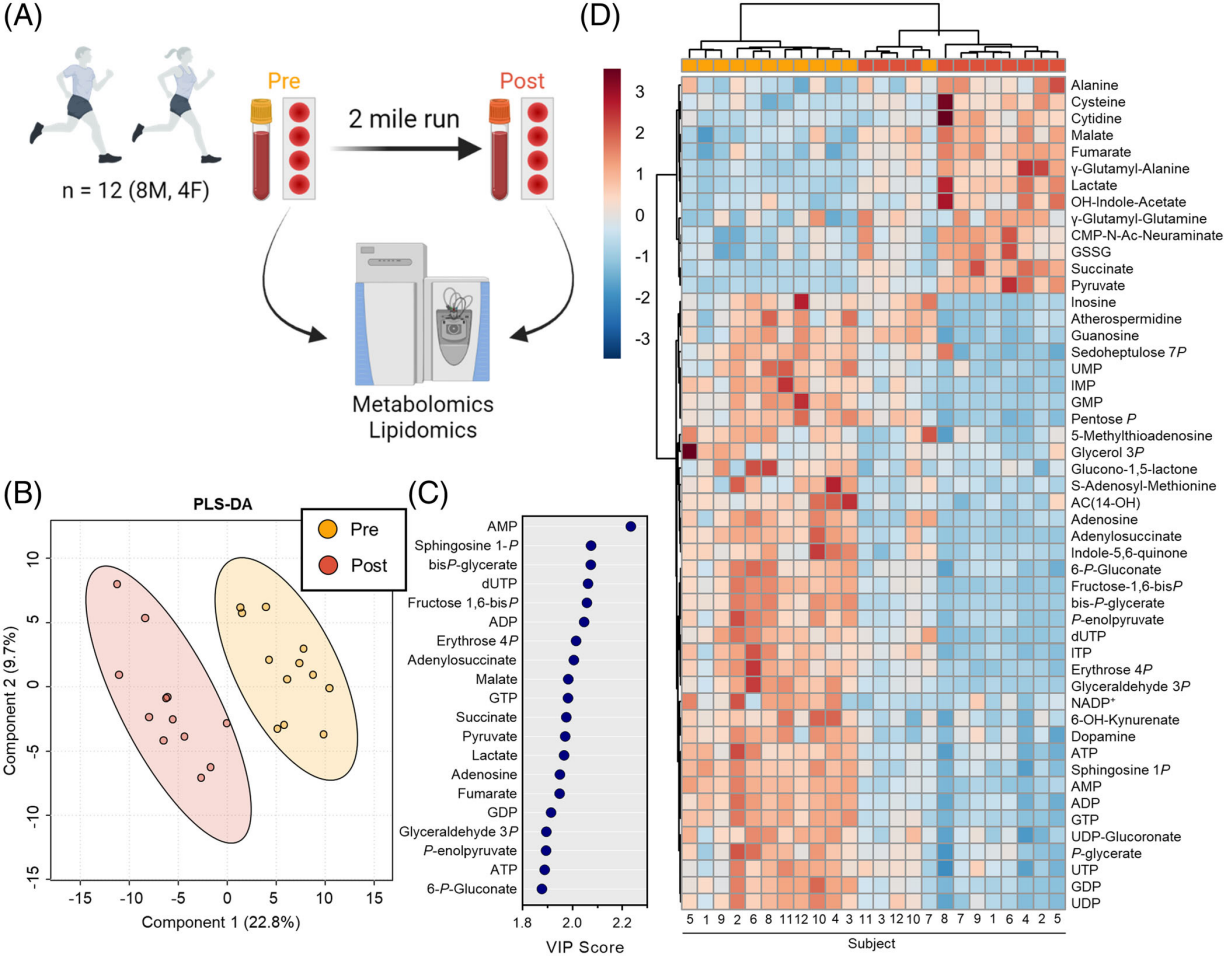
Dried blood spot metabolomics reveals metabolic changes in response to acute running. (A) Dried whole blood was sampled from 12 moderately active individuals (8 male, 4 female) before and after a 2‐mile run and analyzed by mass spectrometry‐based metabolomics and lipidomics. (B) Partial Least Squares‐ Discriminant Analysis (PLS‐DA) distinguishes the samples on the basis of relative metabolite levels with the top 20 metabolites contributing to pattern (Variable Importance in Projection, VIP) plotted in (C). (D) The top 50 significant metabolites by ANOVA were used to hierarchically cluster metabolite patterns and runners

Whole blood samples were obtained from twelve subjects (8 males and 4 females) before and after a 2‐mile run and dried at ambient conditions before subsequent analysis by mass spectrometry‐based metabolomics and lipidomics 3 days later (Figure 1A), providing relative quantitative values for 225 metabolites and 1118 lipids after data curation. Partial Least Squares‐Discriminant Analysis (PLS‐DA) of metabolomics data discerned samples taken before and after the run across the Component 1 axis, which described 22.8% of the variability (Figure 1B). The top 20 metabolites that contributed to this clustering pattern involved intermediates of energy and redox metabolism including glycolysis (bisphosphoglycerate, fructose 1,6‐bisphosphate, glyceraldehyde 3‐phosphate, phosphoenolpyruvate, pyruvate, and lactate), the Tricarboxylic Acid (TCA) Cycle (succinate, fumarate, malate), guanylates and adenylates (GDP, GTP, adenosine, AMP, ADP, ATP), and the Pentose Phosphate Pathway (PPP, 6‐phosphogluconate, erythrose 4‐phosphate) (Figure 1C). Hierarchical clustering of subject samples using the top 50 significant metabolites that changed during the run demonstrated distinct circulating metabolite signatures and was able to distinguish all samples according to time point except for one Pre timepoint sample that clustered together with the Post time point samples, and closest to the respective Pre timepoint from this subject (Figure 1D). Notably, this person was the fastest to complete the trial (sub 6 min/mile pace) and the most trained in this cohort as the only subject to have completed multiple marathons. Thus, the closest relative proximity of these two samples suggests a smaller magnitude of metabolic changes within this subject during the run and might be related to the relatively high training status of this individual with respect to the entire cohort.

### 3.2 Energy metabolite changes in response to acute running

**FIGURE 2:**
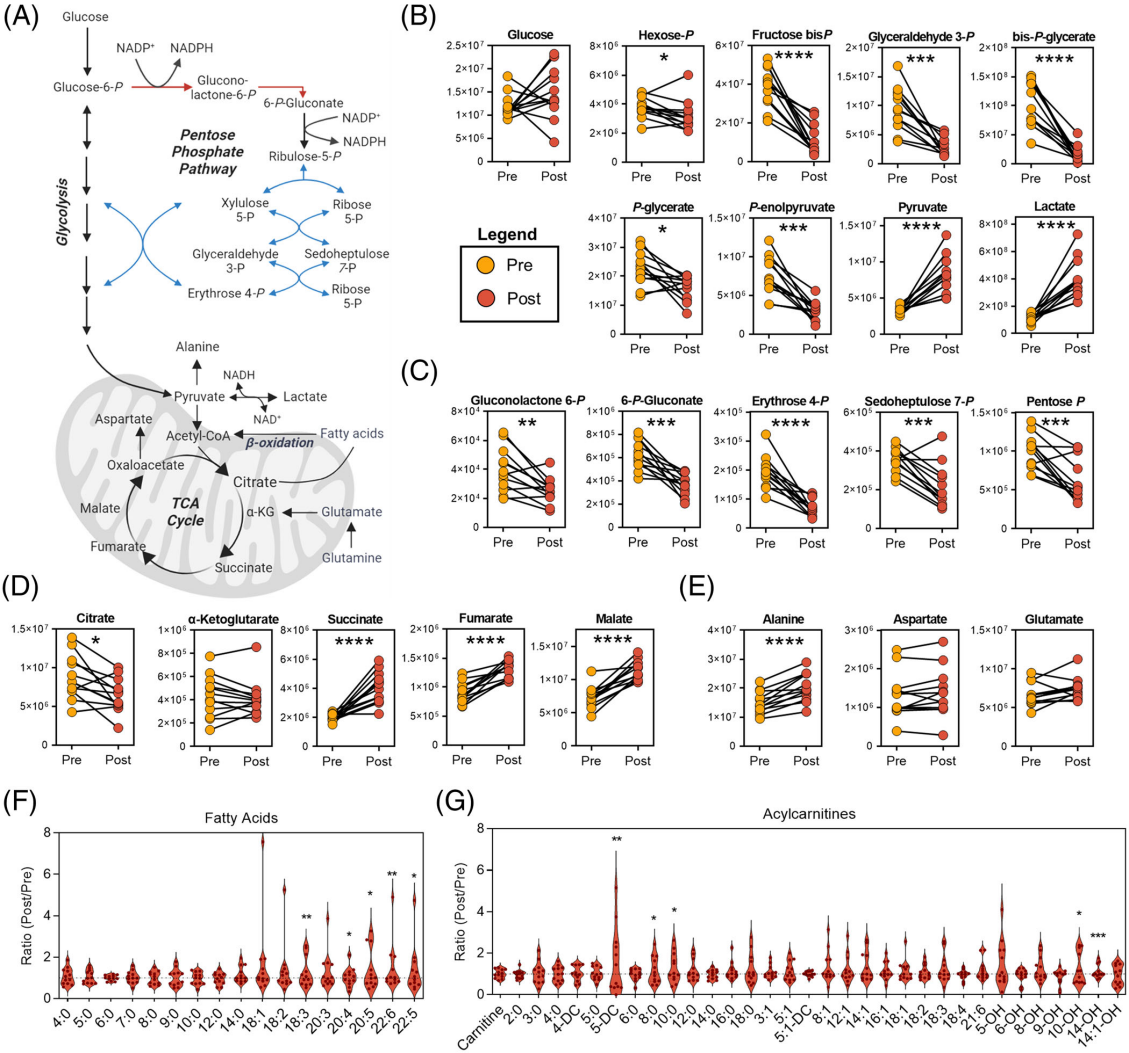
Energy Metabolism. (A) A pathway map for energy generation is shown, along with plots for metabolites in (B) Glycolysis, (C) the Pentose Phosphate Pathway, (D) the Tricarboxylic Acid (TCA) Cycle, (E) corresponding transamination products, (F) free fatty acids, and (G) acylcarnitines. y‐axis values given as peak areas (AU) unless otherwise noted. P‐values for Post/Pre paired comparisons are indicated as * < 0.05, ** < 0.01, *** < 0.001, **** < 0.0001

Interrogation of individual metabolites that distinguished pre‐ and post‐run profiles highlighted significant effects that running elicits on circulating metabolic profiles (Figure 2A). While the mobilization of glucose via glycogenolysis varied amongst subjects, significant glycolytic flux was indicated from steady state measurements by decreased early stage and increased late‐stage glycolytic intermediates, resulting in the accumulation of both pyruvate and lactate (Figure 2B). In similar fashion, intermediates of the PPP were significantly lower after running, suggesting a re‐routing of glucose‐derived carbon towards glycolysis (Figure 2C). This down‐regulation of the PPP likely occurs in red blood cells to fuel late glycolysis in a pH‐dependent process.^13^ Trends in TCA Cycle intermediates were also significant, with lower citrate and higher levels of succinate, fumarate, and malate (Figure 2D). Indeed, release of succinate into the extracellular compartment is also a pH‐dependent process and is coupled with impaired mitochondrial bioenergetics.^35^, ^36^ In contrast, α‐ketoglutarate variably changed along with transamination couple glutamate (Figure 2E). Significant accumulation of alanine, however, suggests elevated alanine aminotransferase (ALT) activity to cope with rapidly accumulating pyruvate and prevention of lactate generation (Figure 2E). In addition to utilization of carbohydrate‐derived carbon to fuel the TCA cycle via pyruvate, fatty acids also provide a significant source of acetyl‐CoA for TCA cycle function. During this run, fatty acid oxidation was highly variable amongst subjects with wide ranges of observed liberated fatty acids (Figure 2F) and acylcarnitines (Figure 2G) in circulation. These results recapitulate previous metabolomics studies on the impact of running^37^ (or other short bouts of high intensity exercise^14^, ^38^) on the circulating metabolome, including activation of glycolysis and concomitant accumulation of lactate, in addition to elevated levels of TCA cycle intermediates.

In addition, exposome agents such as those obtained by diet (e.g., caffeine), substance usage (cotinine, oxyresveratrol), or medication (e.g., ibuprofen) were detected (Supplemental Figure S1). While this study was not powered enough to assess any associated impact between performance and the presence or abundance of these compounds, it would be interesting to expand on the molecular effects of these molecules in an exercise setting considering the established impact they have on red blood cell function,^39^ especially in the case of caffeine^40^, ^41^ and the nicotine‐catabolite, cotinine.^42^

### 3.3 Metabolic profiles return to baseline within 24 hours

**FIGURE 3:**
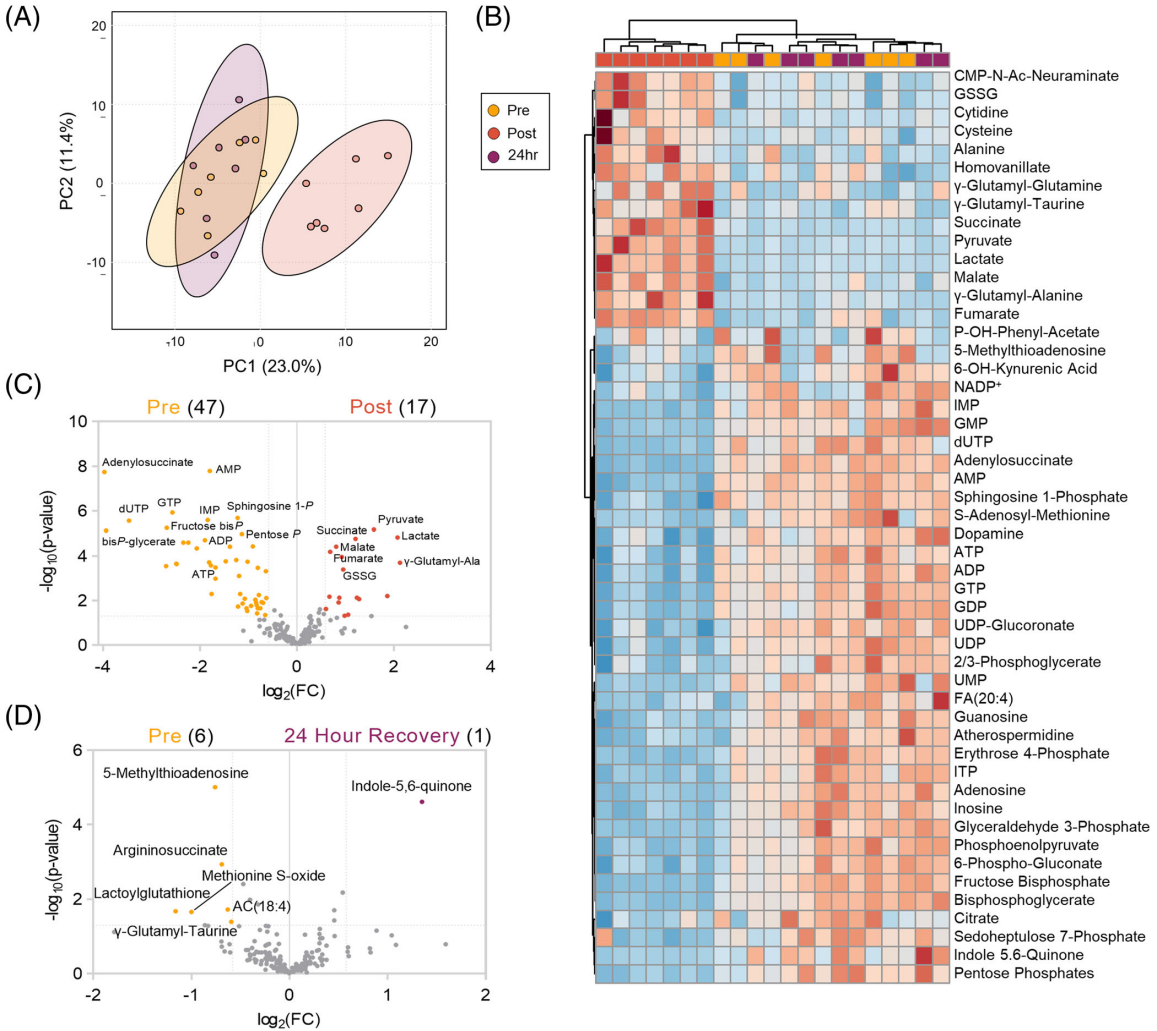
Recovery of metabolic profiles after 24 hours. (A) A PLS‐DA of samples for which a 24‐hour recovery timepoint had also been collected (*n* = 7). (B) Hierarchical clustering of the top 50 significant metabolites by ANOVA. (C) Volcano plot of Pre versus Post run for this cohort, and (D) Pre versus 24‐hour recovery

To demonstrate the ability of this approach to provide longitudinal resolution of metabolic responses to running, we also obtained a sample 24 hours after the run in a subset (*n* = 7) of subjects. PLS‐DA distinctly clustered the post‐run timepoint away from the overlapping samples isolated before the run and 24 hours afterward (Figure 3A). While 47 metabolites or 17 metabolites significantly decreased or increased, respectively, during the run, they predominantly returned to baseline levels within 24 hours (Figure 3B and C). Only 7 metabolites remained significantly changed at this timepoint. Compounds related to redox management (lactoylglutathione, methionine S‐oxide, γ‐glutamyl‐taurine), nitrogen homeostasis (5‐methylthioadenosine, argininosuccinate), and fatty acid oxidation (AC(18:4)) remained lower after 24 hours, while tryptophan catabolite indole‐5,6‐quionine was elevated (Figure 3D). Responses to oxidative stress are a well‐established impact of exercise.^43^ Thus, these compounds may reflect a persistent response to oxidative signaling elicited by the run, either in tissues or in red blood cells which have been reported to accumulate oxidative lesions after exercise.^44^ In this view, persistently lower levels of argininosuccinate and 5‐methylthioadenosine may reflect alterations to nitrogen homeostasis resulting from nitric oxide production to stimulate vasodilation,^45^, ^46^ as well as mediate ongoing purine salvage in red blood cells, which is also modulated by oxidative stress.^47^, ^48^ Finally, higher levels of indole‐5,6‐quinone may indicate an involvement of gut microbiota, which has been increasingly appreciated to contribute to sport performance^49^ as well as motivation to exercise.^50^ While speculative, future studies will be required to disentangle the mechanisms underlying these persistent metabolic changes after running.

### 3.4 Lipidomic measurements reveal two distinct sub‐groups

**FIGURE 4:**
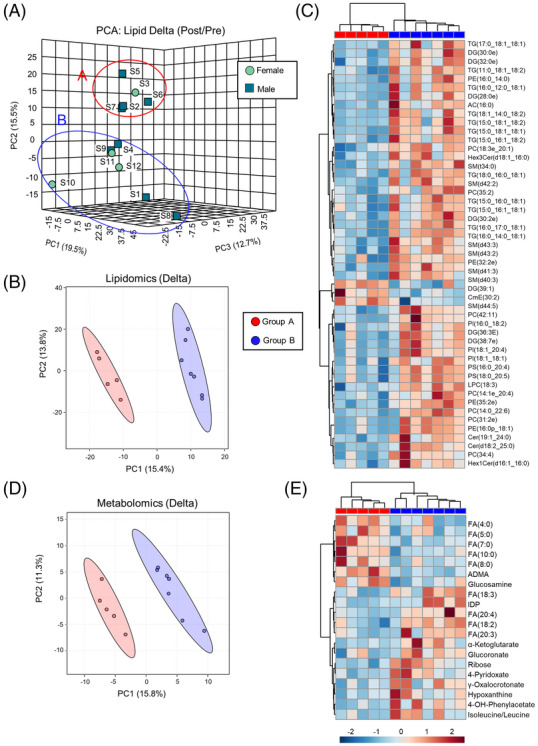
Dried blood spot lipidomics. (A) PCA of delta lipid values (intra‐subject fold change of post/pre) categorized by males and females identifies two distinct subclusters, referred to as Cluster A (red) and Cluster B (blue). (B) PLS‐DA of delta lipid values in Cluster A and B. (C) Hierarchical clustering of the top 50 T‐Test significant lipids between Cluster A and B. (D) PLS‐DA of delta metabolite values in Cluster A and B. (E) Hierarchical clustering of the top 20 T‐Test significant lipids between Cluster A and B

In addition to metabolite profiling, dried blood spot sampling is amenable for lipidomics analysis as well. Samples could not be distinguished by either unsupervised PCA or partially supervised PLS‐DA (Supplementary Figure S2). However, analysis of delta values (intra‐subject normalized to Pre), provided distinct patterns. A linear model adjusting for the covariate of sex was capable of discerning lipids with exercise‐induced changes in a sex‐ and BMI‐dependent fashion despite the small cohort studied here (Supplementary Figure S3). In addition, PCA of delta lipid values identified two distinct, sex‐independent groups (Figure 4A). These groups were further distinguished by PLS‐DA (Figure 4B and Supplementary Figure S4) and hierarchical clustering of the top 50 significant lipids demonstrated a clear pattern of lipid depletion in Cluster A in contrast to accumulation of these same lipids in Cluster B (Figure 4C). Subsequent re‐analysis of metabolomics delta values categorized by these two clusters also revealed distinct patterns by PLS‐DA (Figure 4D and Supplementary Figure S2). Hierarchical clustering of the top 20 significant metabolites between these two clusters highlighted that Cluster A blood samples were enriched for short chain free fatty acids after the run, while samples from Cluster B had elevated levels of long chain free fatty acids (FA(18:2, 18:3, 20:3, 20:4) which were decreased in Cluster A (Figure 4E). In light of the fact that three of the five members of Cluster A were the first 3 to finish the run, and four of the five members reported being highly active, it is interesting to note that this cluster had lower levels of lipids and long chain free fatty acids after the run, a profile of which has been reported as a marker of fitness and associates with performance capacity.^51^ However, steady state measurements cannot discern whether fat is utilized more or mobilized less, and while this study was not designed prospectively nor powered to test this hypothesis, future studies can interrogate fatty acid oxidation using isotope tracers^52^ in combination with dried blood field sampling coupled with respirometry measurements taken in laboratory settings.^51^

### 3.5 Sex specific metabolic responses to running

**FIGURE 5:**
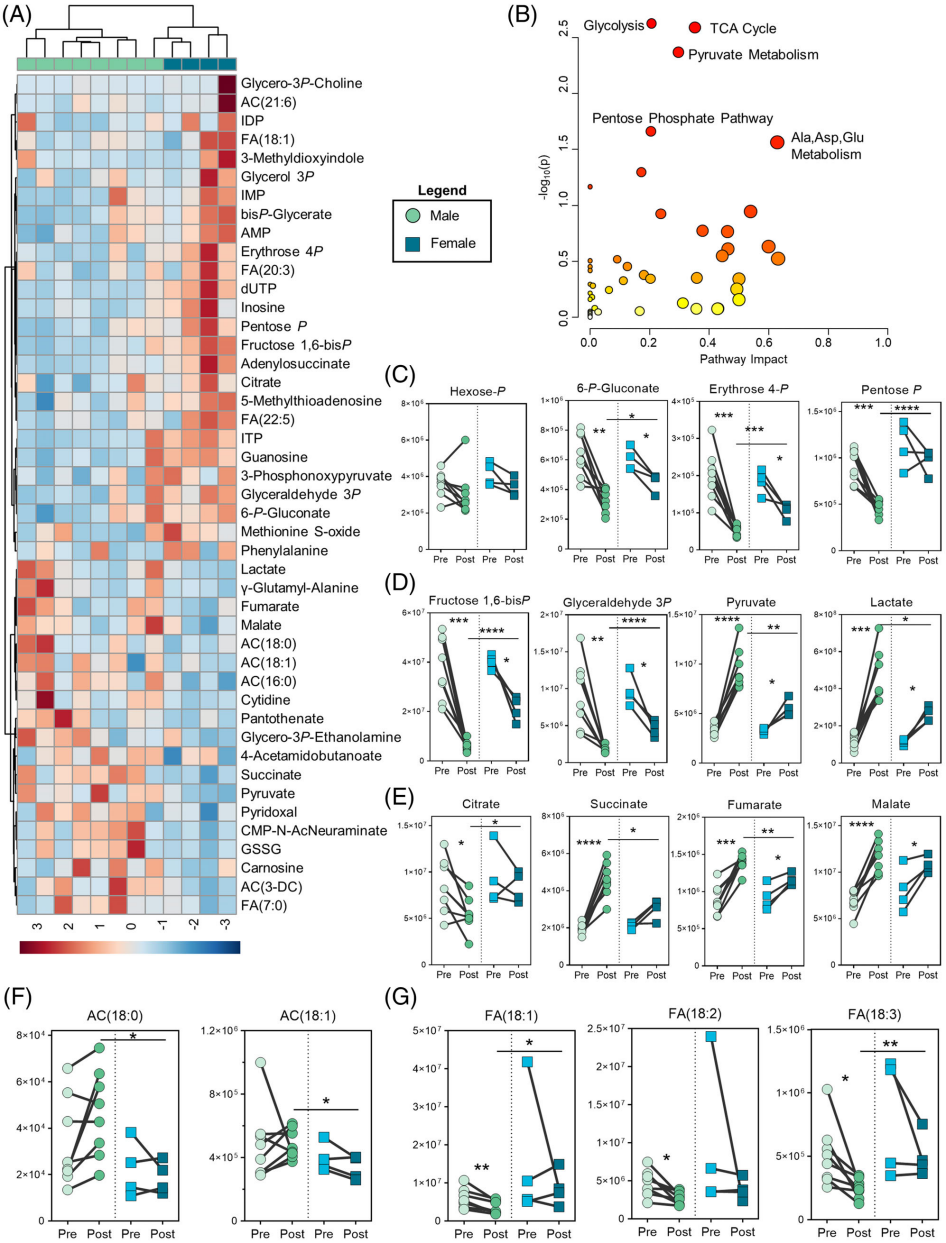
Comparison of male and female responses to acute running. (A) Hierarchical clustering of the top 50 T‐test significant metabolite fold changes (intraindividual Post/Pre normalized). (B) A pathway enrichment map prepared from intraindividual normalized values (Post/Pre) stratified by sex. Metabolites from (C) the PPP, (D) glycolysis, (E) TCA Cycle, (F) acylcarnitines, and (G) free fatty acids plotted at pre and post run values, with males (circles) on the left and females (squares) on the right. y‐axis values given as peak areas (AU). P‐values for Post/Pre comparisons are indicated as * < 0.05, ** < 0.01, *** < 0.001, **** < 0.0001

Hierarchical clustering of metabolite fold‐changes (intrasubject post/pre ratios) was able to separate male from female runners (Figure 5A). Pathway enrichment analysis of these fold‐changes highlighted that sex‐specific patterns pertinent to glycolysis, the TCA Cycle, PPP, and transamination reactions (Alanine, Aspartate, Glutamate Metabolism) (Figure 5B). Analyzing metabolites central to these pathways as a function of sex revealed several significant differences. For instance, while early glycolytic and PPP intermediates significantly decreased during the run, the decrease was smaller in magnitude for female runners who finished with significantly higher levels of these compounds than males (Figure 5C and D). The clear dimorphism observed here may result from the fact that glucose 6‐phosphate dehydrogenase (G6PD), which is the rate‐limiting enzyme of the PPP, is coded by a gene on chromosome X and is expressed higher in women.^53^ In contrast, the accumulation of pyruvate and lactate was higher in males (Figure 5D). These results support reported findings in swimmers, which showed that males generated higher submaximal blood lactate levels than females in a 30‐minute swim test..^54^ In addition, it has been proposed that smaller reductions in glycogen content in type I fibers observed in women after sprinting may also contribute to lower accumulation of blood lactate.^55^

Similar sex‐dependent trends were observed for TCA cycle intermediates citrate, succinate, and fumarate (Figure 5E). Sex dimorphism in carboxylate accumulation is consistent with previous reports describing a lower respiratory exchange ratio in women compared to men, indicating lesser reliance on carbohydrate oxidation to support fuel requirements for exercise.^56^ Tentative explanations for this phenomenon could depend on hematological, muscular or cardiovascular differences. Hematocrit percentages and hemoglobin levels^57^ are sex‐dependent and could contribute to differential reliance on glycolytic versus mitochondrial metabolic pathways via altered oxygen delivery. In addition, dimorphisms in skeletal muscle,^58^ respiratory muscle morphology (females typically have smaller lungs than males even when matched for height^59^, ^60^), and lung geometry,^61^ ultimately impact VO_2_ max (lower in females) and subsequent fatigue resistance.

In addition to carboxylates, males finished the run with higher circulating levels of abundant long chain acylcarnitines including AC(18:0) and AC(18:1) (Figure 5F). In contrast, females finished with higher levels of abundant free fatty acids including FA(18:1) and FA(18:3), which significantly decreased only in males (Figure 5G). These results highlight an increased reliance on fatty acid, rather than glucose oxidation in females, consistent with reported findings.^62^ Female‐predominant elevation in the levels of very‐long chain poly‐ and highly unsaturated fatty acids observed after exercise are notable considering that fatty acid carbon chain length is inversely proportional to the efficiency of fatty acid oxidation in mitochondria,^63^ a phenomenon that is at least in part counterbalanced by the degree of unsaturation.^64^ Activation of endogenous fatty acid desaturases, including those present in red blood cells,^65^ is promoted by oxidant stress. In this view, it is worth noting that fatty acid synthesis and oxidation are processes that rely on the redox equilibrium of the cofactor nicotinamide adenine dinucleotide phosphate (NADP), the production of which is also predominantly dependent on G6PD activity and level. Thus, these observations have clear implications not just in fatty acid homeostasis, but also in redox homeostasis. Since exercise‐induced oxidant stress is a well‐established phenomenon,^43^ and polymorphisms to G6PD are extremely common in humans (∼500 million people suffer to some degree of G6PD deficiency that impacts blood cell metabolism and susceptibility to stress‐induced hemolysis^66^), it is interesting to speculate that sex dimorphism and, perhaps, G6PD deficiency may play an as of yet unappreciated role in exercise performance.

## 4 CONCLUDING REMARKS

Successful optimization of field sampling may help pave the way towards the “democratization” of metabolomics‐guided approaches for exercise interventions, as determined by availability, distribution, and economic considerations. This potential is salient for sports training and exercise performance applications—a global market for blood lactate measurement^67^ alone was valued at $128 M in 2021.^68^ Indeed, in the Olympic spirit of equity and fairness in sports, all athletes, not just elite professionals with economically solid sponsors, should have access to latest generation omics for exercise and training guidance. Similar considerations hold true for the emerging practices of exercise‐based metabolic interventions in fields as diverse as cardiopulmonary diseases (e.g., chronic obstructive pulmonary disease^69^ or pulmonary hypertension^70^) and cancer.^71^, ^72^, ^73^

One main roadblock to the feasibility of remote testing of at home collected blood samples for these emerging medical applications is not only tied to collection and shipment protocols, but also to the intrinsically elevated costs of metabolomics studies. To tackle this issue, we recently developed an ultra‐high‐throughput metabolomics method that enables the analysis of ∼1000 samples/day, making it economically and logistically feasible to scale metabolomics testing for large volumes of samples.^74^ Coupling of dried blood field sampling and ultra‐high‐throughput omics approaches can pave the way for the dissemination of metabolomics from a laboratory science to a more widely applicable discipline; a revolution foretold by early application of metabolomics approaches in the fields of clinical biochemistry^75^ and medical diagnostics and prognostics, especially when coupled to machine learning approaches.^76^ Based on our results, we foresee the feasibility of omics applications that leverage at‐home sample collection in combination with remote training by expert physicians and nutritionists or even simply paired to increasingly common at home electronic training work‐out stations (e.g., for cycling, running or bodybuilding) to enhance physiological responses to these activities and potentially augment or innovate capabilities of real‐time health monitoring wearables.

## AUTHOR CONTRIBUTIONS

FC, TN, and AD designed the study. FC performed exercise tests and collected the samples in the field setting. TN, FC, and AD performed metabolomics analyses; TN and FC performed data analyses and TN prepared figures. All authors contributed to writing the manuscript.

## CONFLICT OF INTEREST

The authors declare that TN and AD are co‐founders of Omix Technologies, Inc. AD is a SAB member for Hemanext Inc, Macopharma, and Forma Therapeutics Inc.

## Supporting information

Supporting Information

Supporting Information

## Data Availability

The data that supports the findings of this study are available in the supplementary material of this article.
