# Supplementary material for: Dried blood spot characterization of sex‐based metabolic responses to acute running exercise
Source: Anal Sci Adv. 2023 Feb 5;4(1-2):37–48. doi: 10.1002/ansa.202200039 (PMC10989637; doi:10.1002/ansa.202200039)
Supplement: Supplementary file 1 — Supporting Information [file ANSA-4-37-s002.pdf]

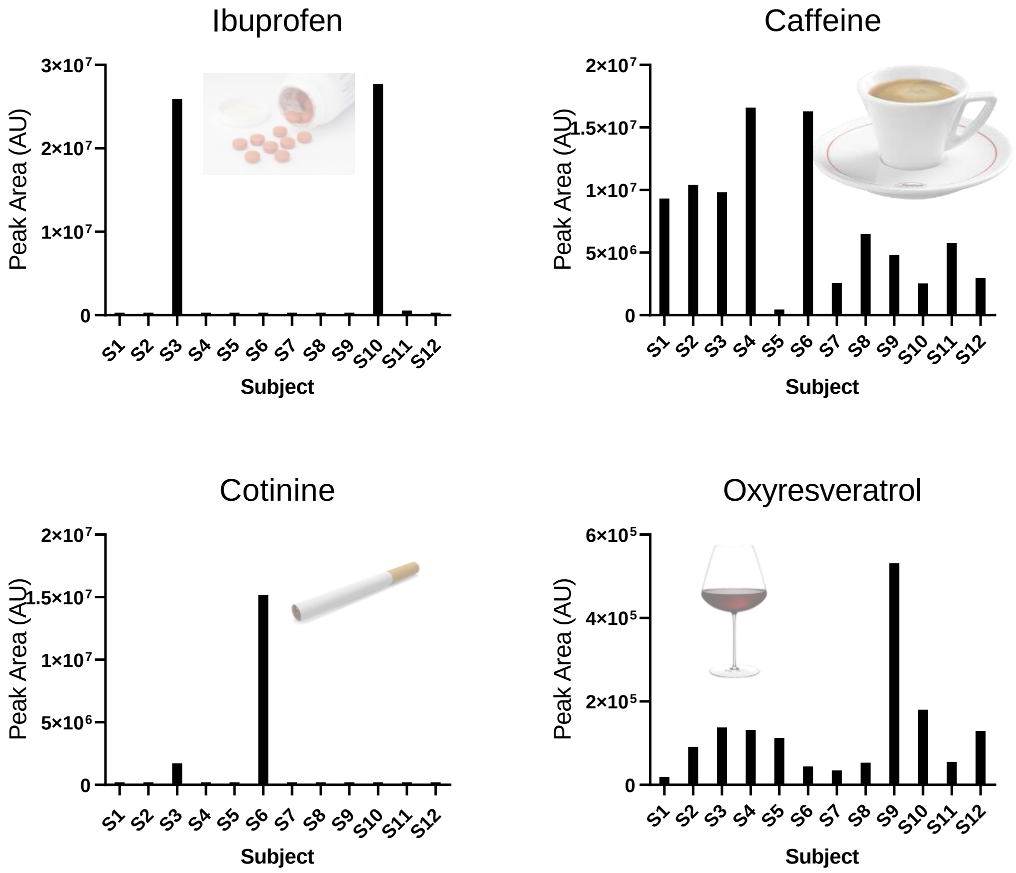

Supplemental Figure 1 Exposome agents.

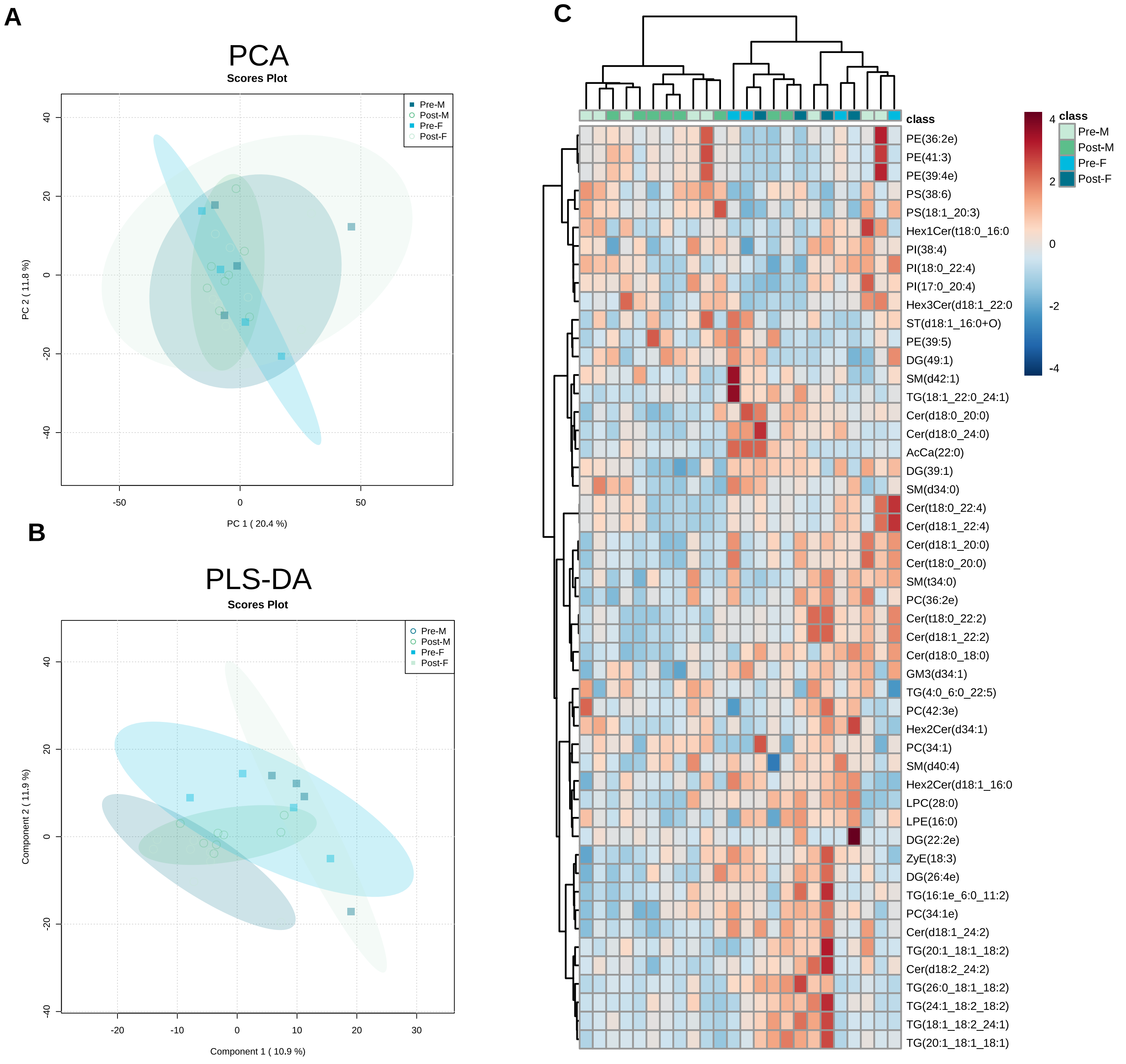

Supplemental Figure 2 Dried blood spot lipidomics. (A) PCA of lipids from Pre and Post time points organized by sex, along with (B) PLS-DA of the same samples. (C) Hierarchical clustering of the 50 lipids with lowest ANOVA score (none were significant, $p < 0.05$).

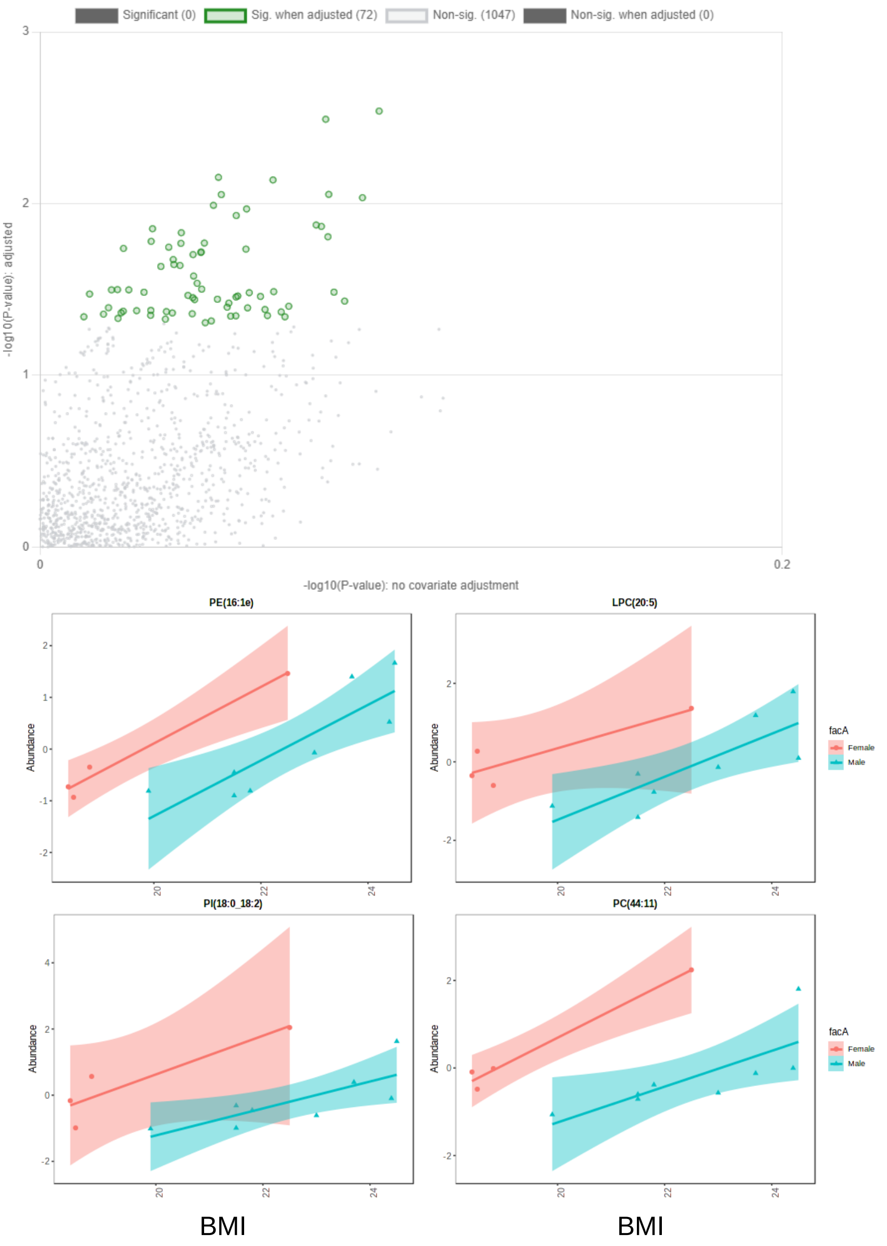

Supplemental Figure 3 Linear Model of Delta Lipid Data as a function of BMI and controlled for Sex.

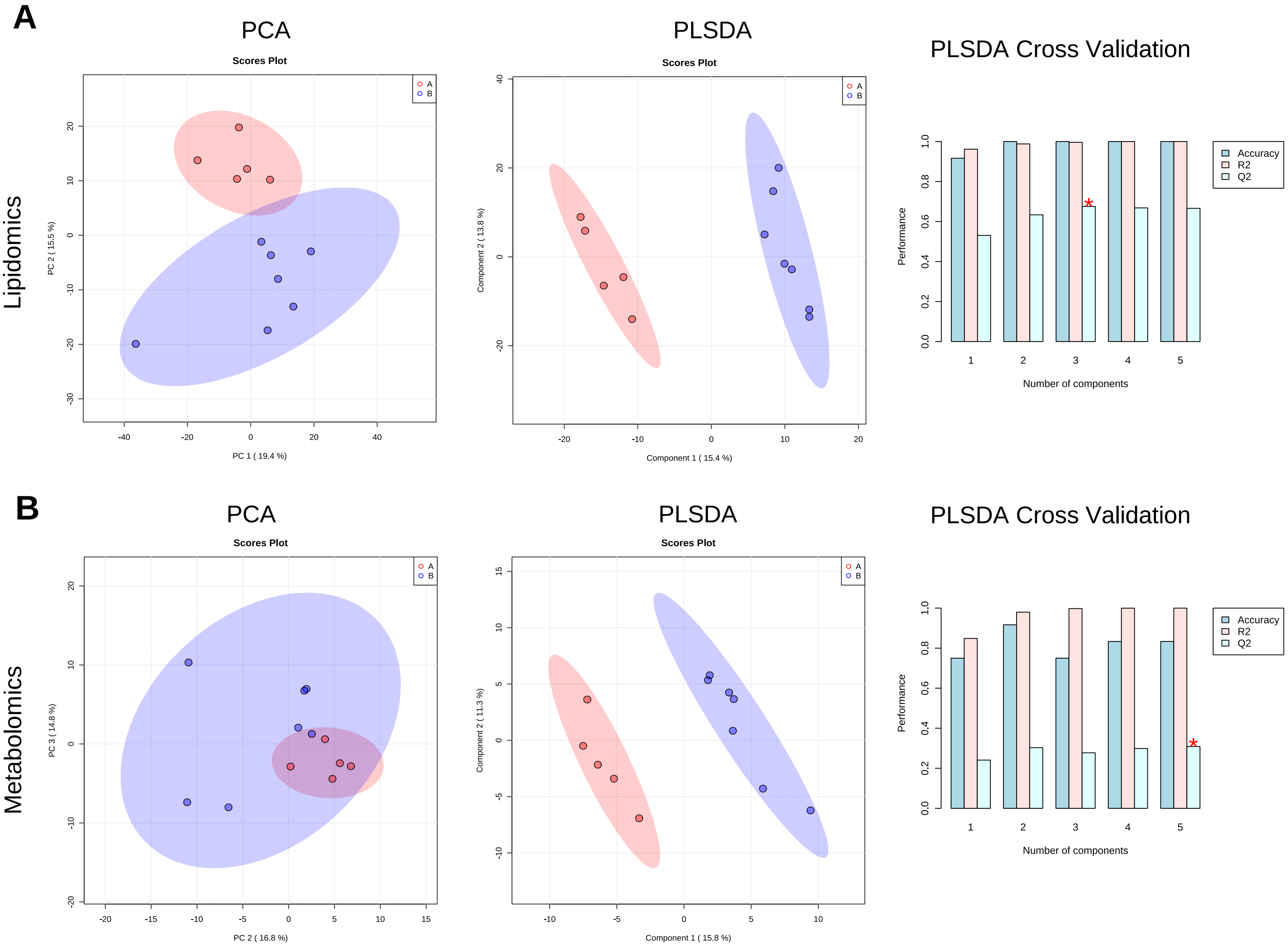

Supplemental Figure 4 Lipidomics and Metabolomics Characterization of two distinct clusters. The PCA, PLS-DA, and PLS-DA Cross validation are shown for the delta (intrasubject post/pre ratio) values in (A) lipidomics and (B) metabolomics datasets.
